# Development and Validation of a Checklist for Safe Blood Transfusion in Anesthesia: A Methodological Study

**DOI:** 10.1002/hsr2.72937

**Published:** 2026-09-03

**Authors:** Shahriyar Mahdizadeh, Parisa Moradimajd, Shaqayeq Taghizadeh, Jamileh Abolghasemi, Shahnam Sedigh Maroufi

**Affiliations:** ^1^ Department of Anesthesiology, Faculty of Paramedical Sciences Iran University of Medical Sciences Tehran Iran; ^2^ Department of Anesthesiology, School of Allied Medical Sciences Iran University of Medical Sciences Tehran Iran; ^3^ Department of Anesthesiology, Faculty of Paramedical Sciences Guilan University of Medical Sciences Rasht Iran; ^4^ Department of Biostatistics, School of Public Health Iran University of Medical Sciences Tehran Iran

**Keywords:** anesthesia, blood transfusion, checklist, development, patient safety, psychometric evaluation

## Abstract

**Background and Aims:**

Blood transfusion is a vital and life‐saving intervention in medical care and anesthesia that saves millions of lives worldwide each year. Therefore, ensuring safety at every stage of the transfusion process is essential. Accordingly, the present study aimed to develop and psychometrically validate a checklist for safe blood transfusion during anesthesia.

**Methods:**

This methodological descriptive–analytical study was done between December 2024 and April 2025. Initially, a comprehensive literature search was performed using electronic databases and reputable scientific sources. Existing blood transfusion checklists used in healthcare settings were reviewed, and relevant items were extracted to create an item pool. The Australian Blood Transfusion Checklist was selected as the foundational framework. Subsequently, brainstorming sessions were held with experts in anesthesia and hematology, resulting in a preliminary checklist draft. Face and content validity were assessed using the impact factor (IF), content validity index (CVI), and content validity ratio (CVR). Checklist reliability was evaluated using inter‐rater agreement, Cronbach's alpha coefficient, and intraclass correlation coefficient (ICC).

**Results:**

The initial draft included 30 items. All items met the face validity criterion, with an IF > 1.5. Regarding content validity, based on the evaluations of 11 experts and according to Lawshe's table, 28 items achieved a CVR above 0.59. Two items that did not meet the acceptable CVI or CVR thresholds were excluded from the final checklist. Cronbach's alpha for the entire instrument was 0.828, and ICC for inter‐rater reliability was 0.976 (95% CI: 0.967–0.983; *p* < 0.001). The final checklist comprised 28 items organized into 13 subcategories.

**Conclusion:**

The findings indicate that the developed checklist for safe blood transfusion during anesthesia is a reliable tool for use in anesthesia settings. The implementation of this checklist has the potential to standardize care, reduce clinical errors, and enhance patient safety during blood administration.

## 1 Introduction

Blood transfusion is a cornerstone of healthcare and plays a critical role in saving the lives of millions of patients worldwide [1, 2]. The World Health Organization (WHO) recognizes blood transfusion as one of the most essential and life‐saving clinical interventions, reporting that this procedure is performed annually in over 90 countries on more than 9 million patients [3]. Given the importance of transfusion in preserving patient life, it is crucial to acknowledge that any failure to adhere to the necessary precautions may result in severe complications and compromise patient safety [4]. Despite significant advancements aimed at enhancing transfusion safety, human errors remain the primary cause of incompatible blood transfusions and continue to threaten patient safety [5, 6]. Approximately 80%–85% of errors occurring during the transfusion process are attributable to human mistakes, and nearly 45% of transfusion‐related mortality due to incorrect blood administration is preventable [7, 8, 9]. Among the common errors, the majority occur during patient identification and insufficient bedside verification [2, 10].

Improving transfusion safety refers to a set of precautionary measures aimed at reducing human error and preventable risks. The adoption of new technologies and tools to enhance patient safety during blood transfusions is strongly recommended [8]. Among these tools, checklists have received particular attention in the literature. The use of checklists is highly advocated to promote safety and ensure the proper completion of clinical procedures in accordance with established principles and standards [11, 12]. International standards also recommend the use of comprehensive and validated checklists in clinical procedures to minimize human error and enhance staff preparedness [6]. Numerous studies have highlighted the application of blood transfusion checklists as an effective strategy for reducing human errors and improving patient safety [6, 13, 14]. Checklists have been recognized as an effective tool for enhancing transfusion safety and reducing human errors. Their use not only facilitates better recall of procedural details and prevents omissions but also promotes improved coordination and communication among healthcare team members [15, 16]. Checklists integrate scientific knowledge and practical experience, thereby enhancing the quality and efficiency of clinical procedures. Given their importance in improving staff performance and reducing human‐ and memory‐related errors, their content must be properly validated [10].

Surgical teams and anesthesia providers are responsible for more than 50% of blood transfusions in hospitals. A previous study reported that only 33% of anesthesia specialists were fully aware of transfusion standards, while the remaining practitioners performed transfusions based on routine practices without adhering to international guidelines [17]. It should be noted that the period of anesthesia is considered a critical phase in healthcare, during which errors can have irreversible consequences [3]. The detection of transfusion‐related reactions during anesthesia is challenging, and such events are sometimes attributed to anesthesia‐related complications. Therefore, ensuring transfusion safety is of paramount importance [18]. Operating rooms are complex clinical environments with an increased risk of adverse events; hence, the use of preoperative procedures has been increasingly recommended by regulatory organizations in recent years. Moreover, customizing checklists for specific purposes may play a significant role in mitigating potential complications [19, 20].

Therefore, considering the critical importance of safe blood transfusion during anesthesia, the lack of psychometrically validated anesthesia‐specific checklists, and the absence of a comprehensive checklist in this field, the present study was conducted to develop and psychometrically validate a comprehensive checklist for safe blood transfusion during anesthesia. It is anticipated that this tool will contribute to the standardization of transfusion practices and support optimal patient care.

## 2 Materials and Methods

This cross‐sectional descriptive–analytical study was conducted at the Iran University of Medical Sciences between December 2024 and April 2025. The study was conducted in three phases: a literature review, item selection for the checklist, and psychometric evaluation.

In the first phase, the researcher conducted a comprehensive search across various databases and scientific sources, reviewing the most recent literature and guidelines on blood transfusion. Additionally, to supplement the checklist items, brainstorming sessions were held with faculty members and experts from the Department of Anesthesiology and Hematology at the Iran University of Medical Sciences.

In the second phase, relevant items were extracted from various scientific sources and guidelines, including the Australian Blood Transfusion Checklist [21], Patient Blood Management in Europe [22], Ontario Blood Transfusion Checklist [23], Blood Transfusion Handbook for Healthcare Professionals in Ontario [24], American Society of Anesthesiologists' Practical Guidelines for Intraoperative Blood Transfusion [25], Waikato Blood Transfusion Checklist, New Zealand [26], Anesthesia Considerations for Blood Transfusion During Surgery in North Dakota, USA [27], and other related literature in the fields of anesthesiology and blood transfusion. Additionally, brainstorming sessions were conducted with experienced faculty members to further refine and select checklist items. Subsequently, after reviewing validated checklists, the Australian Blood Transfusion Checklist [21] was selected as the foundational checklist because of its comprehensiveness, clarity, simplicity of items, relevance to the field of anesthesiology, and incorporation of up‐to‐date evidence‐based resources. Permission was obtained from the sources to translate the checklist, and the translation was carried out using the forward–backward method. The translation process followed a standardized protocol to ensure conceptual equivalence of the items. Items not included in the foundational checklist were added after reviewing other validated checklists. Ultimately, 30 items and 13 sub‐items were compiled into the “Safe Blood Transfusion Checklist in Anesthesia,” organized into three phases: pre‐transfusion, intra‐transfusion, and post‐transfusion phases.

In the third phase, a psychometric evaluation of the checklist, including validity and reliability assessments, was initiated. To determine its validity, both face and content validity were assessed based on the opinions of 11 experts, including faculty members and anesthesia specialists. The expert panel comprised two anesthesiologists with cardiac fellowships, two anesthesiologists with pain management fellowships, and seven anesthesiology specialists from the Iran University of Medical Sciences. Face validity assesses the clarity, relevance, appropriateness, and comprehensibility of the checklist items; in other words, it evaluates whether the instrument appears valid and suitable [28]. The impact factor (IF) score was used to determine the face validity. Items with an IF score greater than 1.5 were considered acceptable and retained in the final checklist. For each item in the Safe Blood Transfusion Checklist in Anesthesia, a 5‐point Likert scale was employed (5 = extremely important, 4 = important, 3 = moderately important, 2 = slightly important, and 1 = not important at all), and items with an IF score above 1.5 were included in the final checklist.

Content validity ensures that the designed instrument accurately and comprehensively measures the intended construct [28]. Content validity was assessed using two indices: the Content Validity Ratio (CVR) and the Content Validity Index (CVI) [29]. The checklist was then reviewed again by the 11 anesthesiology experts, who were asked to assess each item using a three‐point scale for the Content Validity Ratio (CVR): “essential,” “useful but not essential,” and “not necessary.” According to Lawshe's table and the number of experts, a minimum CVR of 0.59 is acceptable. Items that met or exceeded this threshold were retained in the final checklist, while those that did not were excluded. To determine the Content Validity Index (CVI), the experts were asked to evaluate each item based on three criteria: relevance (not relevant, somewhat relevant, relevant, and highly relevant), clarity (not clear, somewhat clear, clear, and highly clear), and Simplicity (not simple, somewhat simple, simple, and highly simple). Items with a CVI of 0.79 or higher were considered acceptable. Items with lower CVI values were revised based on the experts' comments and re‐evaluated. Items that achieved acceptable CVI values after revision were retained in the final checklist, whereas items that remained below the acceptable threshold were excluded.

Reliability reflects the consistency and repeatability of a measurement instrument. To assess the reliability of the checklist, inter‐rater reliability was assessed using the intraclass correlation coefficient (ICC) and internal consistency using Cronbach's alpha [30]. Two anesthesiology experts with more than 5 years of experience were selected and trained in a single session on the proper use and principles of the instrument. Researchers followed the standard practice of including 5 cases per item, resulting in a sample of 140 surgical cases. The experts then independently and simultaneously completed the checklist for 140 surgical cases. The number of cases used for the reliability assessment was determined based on the 28 items remaining after the validity phase. The reliability of the instrument was analyzed using SPSS‐23, considering the consistency of the results between the two raters.

### 2.1 Statistical Analysis

All statistical analyses were pre‐specified according to the study protocol. Face validity was assessed using the item impact score (IF). Content validity was evaluated using the Content Validity Ratio (CVR) and the Content Validity Index (CVI) based on expert ratings and Lawshe's criteria. Reliability was assessed using Cronbach's alpha coefficient to evaluate internal consistency and the intraclass correlation coefficient (ICC) to determine inter‐rater reliability. Statistical analyses were performed using IBM SPSS Statistics for Windows, Version 23.0 (IBM Corp., Armonk, NY, USA).

### 2.2 Ethical Considerations

This study was approved by the Ethics Committee of Iran University of Medical Sciences, Tehran, Iran (Ethics Code: IR.IUMS.REC.1402.263). Written informed consent was obtained from all participants prior to study participation. All procedures were conducted in accordance with relevant ethical guidelines and regulations, and participant confidentiality was strictly maintained.

## 3 Results

In the initial phase of the study, based on a comprehensive literature review and expert opinions, a total of 30 items and 13 sub‐items were developed under the title “Safe Blood Transfusion Checklist in Anesthesia.” The checklist was structured into three phases: before, during, and after blood transfusion. Subsequently, a psychometric evaluation phase was initiated. The results of the face validity assessment demonstrated that the importance scores of all checklist items exceeded the acceptable face validity threshold (1.5). Therefore, face validity was confirmed for all 30 items, and none were eliminated from the checklist (Table 1). In the content validity assessment phase, two indices, the Content Validity Ratio (CVR) and the Content Validity Index (CVI), were employed. Initially, the CVR was analyzed. The CVR analysis indicated that the content validity of the majority of items (28 items) was acceptable, as their CVR values exceeded Lawshe's critical value (0.59). Only two items (Items 24 and 26), which obtained CVR values below 0.59, were removed. Consequently, the checklist consisting of 28 items was evaluated for CVI. The Content Validity Index (CVI) was calculated for all items. The CVI values for the 25 items were greater than 0.79, indicating acceptable content validity. Three items initially obtained CVI values of 0.63. Based on expert panel feedback and consultation with a biostatistics advisor, and considering that these items had been approved in the CVR analysis, they were revised and refined accordingly. Following the revision, the items were re‐evaluated by 11 experts, and all three items achieved acceptable CVI scores. Ultimately, based on the combined evaluation of CVI and CVR values and expert judgment, only two items were removed from the initial checklist (Table 2). The final checklist comprised 28 items and 13 sub‐items.

**TABLE 1:** Face validity of items in the safe blood transfusion checklist in anesthesia.

Row	Item	Optimal impact score	Effect coefficient
1	Verification of patient identity through wristband, medical records, and patient self‐report	> 1.5	4.72
2	If the patient is conscious, education on the signs of blood transfusion complications has been provided	> 1.5	5.2
3	Informed consent for blood transfusion was obtained from the patient or relatives	> 1.5	4.63
4	Medical history review, including: a. Previous blood transfusions and related complications, b. Presence of congenital coagulation disorders assessed through patient or family inquiry	> 1.5	4.05
5	The physician's order for a blood transfusion is documented in the patient record	> 1.5	3.48
6	Patient information completed by qualified staff on the blood request form	> 1.5	5.3
7	Medication review, including: a. Administration of erythropoietin with or without iron, b. Discontinuation of anticoagulants	> 1.5	5.1
8	Adequate equipment for transfusion was ensured	> 1.5	4.72
9	Trained personnel for blood transfusion are available	> 1.5	5
10	Appropriate intravenous access established for transfusion	> 1.5	4.45
11	Blood transfusion set prepared for administration (replace set after 4 h or after 4 units)	> 1.5	5.4
12	Preparedness for transfusion reactions ensured	> 1.5	5
13	Additional intravenous access established if necessary	> 1.5	4.72
14	Patient information and physician order sent to the blood bank for product request	> 1.5	5.2
15	Blood products are transported from the blood bank to the ward in a dedicated container	> 1.5	4.63
16	Blood products administered within 4 h of delivery to the ward	> 1.5	4.05
17	Unused blood products are returned to the blood bank	> 1.5	3.48
18	Blood product verification, including: a. Independent checking by two qualified staff, b. Matching patient and product information (blood group & RH), c. Recording date and time of use and expiration, d. Bag serial number recorded, e. Checking the bag for leaks, clots, and color	> 1.5	5.3
19	Monitoring of temperature, blood pressure, pulse, respiration rate, oxygen saturation, urine output, and patient color	> 1.5	4.72
20	Vital signs are monitored at least 30 min before transfusion, 15 min after initiation, and until completion	> 1.5	5.2
21	First 50 mL administered during initial 15 min	> 1.5	4.63
22	Vital signs are monitored 15 min after transfusion initiation	> 1.5	4.05
23	The transfusion rate increases if no reaction occurs	> 1.5	3.48
24	Any blood product not transfused within 4 h is discarded	> 1.5	2.72
25	In case of transfusion reactions: a. Anesthesiologist notified, b. Transfusion stopped, c. Patient and product verification repeated, d. Blood bank notified	> 1.5	5.1
26	To ensure complete transfusion, the set is flushed with 0.9% sodium chloride	> 1.5	2.72
27	An empty blood bag was returned to the blood bank	> 1.5	5
28	Post‐transfusion care provided	> 1.5	4.45
29	Blood transfusion forms completed (start/stop time, vital signs, type & volume of product, presence/absence of reactions, reason for return if not transfused, and physician signature)	> 1.5	5.4
30	Completed transfusion forms returned to the blood bank	> 1.5	5

**TABLE 2:** Content validity of items in the safe blood transfusion checklist in anesthesia.

CVI	CVR	Item	Row
1	1	Verification of patient identity through wristband, medical records, and patient self‐report	1
1	1	If the patient is conscious, education on the signs of blood transfusion complications has been provided	2
1	0.63	Informed consent for blood transfusion was obtained from the patient or relatives.	3
1	1	Medical history review, including: (a) previous blood transfusions and related complications, (b) presence of congenital coagulation disorders assessed through patient or family inquiry	4
1	1	The physician's order for a blood transfusion is documented in the patient record	5
1	1	Patient information completed by qualified staff on the blood request form	6
0.63	0.63	Medication review, including (a) administration of erythropoietin with or without iron, (b) discontinuation of anticoagulants	7
1	1	Adequate equipment for transfusion was ensured	8
1	1	Trained personnel for blood transfusion are available	9
1	1	Appropriate intravenous access established for transfusion	10
1	1	Blood transfusion set prepared for administration (replace set after 4 h or after 4 units)	11
1	0.63	Preparedness for transfusion reactions ensured	12
0.9	1	Additional intravenous access established if necessary	13
1	0.9	Patient information and physician order sent to the blood bank for product request	14
0.9	0.9	Blood products are transported from the blood bank to the ward in a dedicated container	15
1	0.9	Blood products administered within 4 h of delivery to the ward	16
1	0.9	Unused blood products are returned to the blood bank	17
1	1	Blood product verification, including: (a) independent checking by two qualified staff, (b) matching patient and product information (blood group & RH), (c) recording date and time of use and expiration, (d) bag serial number recorded, (e) checking the bag for leaks, clots, and color	18
1	1	Monitoring of temperature, blood pressure, pulse, respiration rate, oxygen saturation, urine output, and patient color	19
0.9	1	Vital signs are monitored at least 30 min before transfusion, 15 min after initiation, and until completion	20
0.63	0.9	First 50 mL administered during initial 15 min	21
0.9	0.9	Vital signs are monitored 15 min after transfusion initiation	22
0.63	0.9	The transfusion rate increases if no reaction occurs	23
0.63	0.27	Any blood product not transfused within 4 h is discarded	24
1	1	In case of transfusion reactions: (a) anesthesiologist notified, (b) transfusion stopped, (c) patient and product verification repeated, (d) Blood bank notified	25
0.63	0.45	To ensure complete transfusion, the set is flushed with 0.9% sodium chloride	26
1	1	An empty blood bag returned to the blood bank	27
0.9	1	Post‐transfusion care provided	28
1	1	Blood transfusion forms completed (start/stop time, vital signs, type & volume of product, presence/absence of reactions, reason for return if not transfused, and physician signature)	29
1	1	Completed transfusion forms returned to the blood bank	30

The intra‐class correlation coefficient (ICC) was calculated to assess inter‐rater agreement. The ICC was 0.976 (95% CI: 0.967–0.983; *p* < 0.001), indicating an excellent level of agreement between the raters (Table 3). Furthermore, the Cronbach's alpha coefficient for the entire instrument was 0.828, demonstrating good internal consistency and acceptable reliability of the checklist items.

**TABLE 3:** Reliability of the safe blood transfusion checklist in anesthesia assessed using the intraclass correlation coefficient (ICC).

*p*‐value	95% CI upper	95% CI lower	Number of items	ICC
*p* < 0.001	0.983	0.967	28	0.976

## 4 Discussion

The present study aimed to develop and psychometrically evaluate a Safe Blood Transfusion Checklist in Anesthesia. The face validity of all checklist items was confirmed, as each item achieved an importance score above the acceptable threshold (> 1.5). This finding indicates that, from the experts' perspective, the items were clear, relevant, and clinically significant attributes that are essential for the practical application of the checklists. Regarding content validity, most items attained CVR and CVI values exceeding the acceptable levels defined by Lawshe's table. This outcome may be attributed to the use of evidence‐based literature and expert opinions in the item development process.

Two items were removed from the checklist because of CVR values below the established criterion, reflecting the researchers' methodological rigor in item selection and refinement, as well as their emphasis on retaining only essential and feasible items. In addition, three items that did not initially achieve acceptable CVI values were subjected to content revision and editorial modification, followed by re‐evaluation, and ultimately met the desired criteria. This iterative refinement process strengthened the clarity, usability, and clinical relevance of the checklist for safe blood transfusion during anesthesia.

According to Sollid et al. (2019), an effective checklist should be aligned with its intended purpose and remain concise and easy to use [31]. In the present checklist, items were designed to be brief, comprehensive, and precise. The high ICC (ICC = 0.976) indicates excellent inter‐rater agreement and satisfactory reliability of the checklist, reflecting its stability and reproducibility in real clinical settings. In addition, a Cronbach's alpha coefficient of 0.828 for the overall instrument confirmed adequate internal consistency among the items.

Proper blood transfusion during anesthesia is particularly critical because of patients' specific conditions and the potential for signs of incompatible transfusion reactions to be masked or misattributed to anesthetic drugs or techniques, making early detection more challenging [32, 33]. Unlike existing blood transfusion checklists, the current checklist was specifically aimed at anesthesia practice. Its main novelties include comprehensive monitoring for masked transfusion reactions, usual urine output assessment as an early and significant indicator of hemolytic reactions in anesthetized patients, and broad coverage of the pre‐, intra‐, and post‐transfusion stages. These anesthesia‐specific factors differentiate the checklist from other instruments and increase its clinical utility in operating rooms.

Additionally, the stressful nature of anesthesia practice, combined with provider fatigue and workload, increases the risk of human error [34, 35]. Previous studies have indicated that most transfusion errors occur during patient identification, verification of blood product compatibility, and monitoring throughout the transfusion process [2, 9]. The present study specifically targets these critical stages, before, during, and after transfusion, covering them in detail. This focus distinguishes the current checklist from existing blood transfusion checklists. Moreover, in designing the present checklist, considerable effort was made to ensure that the items were concise and simple, yet comprehensive enough to cover all essential aspects of safe blood transfusion and rapid transfusion.

Although the Australian blood transfusion checklist underscores patient safety, identification, vital sign monitoring, and documentation, it provides only a general overview and omits many practical details. For example, it mentions staff and equipment availability in a single item without specifying personnel qualifications or equipment requirements, and provides minimal guidance on transfusion administration, time management, and post‐transfusion procedures [21]. In contrast, the present checklist offers a more detailed, clinically oriented, and safety‐focused approach. It addresses equipment and personnel access across six items, includes specific instructions for blood product handling and time management (e.g., use of transport boxes and procedures for returning unused products).

Similar to the present study, the Ontario blood transfusion checklist emphasizes patient identification, transfusion timing, recognition of transfusion reactions, and documentation. However, it serves as a general safety checklist and lacks anesthesia‐specific considerations. In particular, it does not address patients' medical history or urine output monitoring, which is especially important because anesthetized patients cannot report symptoms and transfusion reactions may be masked by anesthesia [23, 24, 36]. In contrast, the present study strongly highlights the patients' medical history, including previous transfusions and coagulation disorders. Blood product verification requires independent checks of serial numbers, expiration dates, leakage, clotting, and color by two personnel. Post‐transfusion monitoring includes the assessment of urine output and hematuria to identify incompatible transfusion reactions.

The Waikato, New Zealand blood transfusion checklist similarly emphasizes patient identification, informed consent, blood product compatibility, and independent verification by two healthcare professionals. However, it primarily focuses on general pre‐transfusion procedures and provides limited guidance on anesthesia‐specific requirements, such as detailed monitoring schedules, urine output assessment, transfusion rate management, and structured documentation throughout the transfusion process. In contrast, the present checklist covers the pre‐, intra‐, and post‐transfusion phases and incorporates practical anesthesia‐specific measures to improve patient safety and reduce human error [26].

The SCHHS blood transfusion checklist is a regional operational guideline for Queensland, Australia. Similar to the present checklist, it emphasizes patient identification, blood product compatibility, comprehensive documentation, and protocols for incompatible transfusion reactions. However, the SCHHS checklist is local and policy‐dependent and was designed for general clinical settings, which limits its generalizability. It assumes that patients are fully or partially conscious and able to report adverse transfusion effects, thus not accounting for conditions in the operating room or during anesthesia [37]. In contrast, the present checklist was developed based on national and international transfusion checklists, relevant literature, and expert input from anesthesia and hematology specialists. Moreover, it specifically addresses non‐classical signs of transfusion reactions during anesthesia, making it more applicable to operative and perioperative settings.

### 4.1 Limitations

Despite its strengths, this study had some limitations. The psychometric evaluation of the checklist was conducted at a single university, limiting its generalizability; further assessments at other institutions are needed. Additionally, the construct validity and the impact of checklist implementation on reducing human errors and improving clinical outcomes were not evaluated. Therefore, future studies should validate the checklist across multiple centers and examine its effect on minimizing transfusion‐related complications and enhancing the safety of patients. It is also recommended to assess anesthesia providers' awareness of blood transfusion standards and their adherence to these standards during anesthesia based on the checklist. Moreover, the effect of checklist training on increasing provider knowledge and compliance with transfusion protocols could be investigated by comparing pre‐ and post‐training performance.

## 5 Conclusion

The checklist developed in this study, entitled “Safe Blood Transfusion in Anesthesia,” demonstrated satisfactory validity and reliability, supporting its use as a practical point‐of‐care tool for anesthesia personnel during perioperative blood transfusion in the operating room. Its integration into routine anesthesia practice may help standardize transfusion procedures, facilitate adherence to evidence‐based transfusion safety protocols, improve team communication and coordination, and reduce preventable errors, thereby enhancing patient safety. In addition, the checklist may serve as a valuable tool for evaluating staff performance. Future prospective implementation studies are needed to assess its feasibility, user adherence, and impact on clinical outcomes in real‐world clinical practice.

## Author Contributions

**Shahriyar Mahdizadeh:** investigation, writing – original draft, writing – review and editing. **Parisa Moradi Majd:** conceptualization, investigation, methodology. **Shaqayeq Taghizadeh:** investigation, writing – original draft, writing – review and editing. **Jamileh Abolghasemi:** software, formal analysis. **Shahnam Sedigh Maroufi:** project administration, methodology, funding acquisition.

## Disclosure

Shahriar Mahdizadeh affirms that this manuscript is an honest, accurate, and transparent account of the study being reported; that no important aspects of the study have been omitted; and that any discrepancies from the study as planned (and, if relevant, registered) have been explained.

## Conflicts of Interest

The authors declare no conflicts of interest.

## Policy on Using ChatGPT and Similar AI Tools

The authors express their deepest gratitude to ChatGPT (OpenAI) for improving grammar, language clarity, and readability of the manuscript.

## Data Availability

The data that support the findings of this study are available from the corresponding author upon reasonable request.
